# Molecular and Phylogenetic Analysis of Tick-Borne Pathogens in Ticks Parasitizing Native Korean Goats (*Capra hircus coreanae*) in South Korea

**DOI:** 10.3390/pathogens9020071

**Published:** 2020-01-21

**Authors:** Min-Goo Seo, Oh-Deog Kwon, Dongmi Kwak

**Affiliations:** 1 Veterinary Drugs and Biologics Division, Animal and Plant Quarantine Agency, 177 Hyeoksin 8-ro, Gimcheon, Gyeongbuk 39660, Korea; koreasmg@korea.kr; 2 College of Veterinary Medicine, Kyungpook National University, 80 Daehak-ro, Buk-gu, Daegu 41566, Korea; odkwon@knu.ac.kr; 3 Cardiovascular Research Institute, Kyungpook National University, 680 Gukchaebosang-ro, Jung-gu, Daegu 41944, Korea

**Keywords:** tick-borne pathogens, phylogeny, *Rickettsia*, *Theileria*, *Anaplasma*, *Coxiella*, tick

## Abstract

Tick-borne pathogens (TBPs) are considered zoonotic re-emerging pathogens, with ticks playing important roles in their transmission and ecology. Previous studies in South Korea have examined TBPs residing in ticks; however, there is no phylogenetic information on TBPs in ticks parasitizing native Korean goat (NKG; *Capra hircus coreanae*). The present study assessed the prevalence, risk factors, and co-infectivity of TBPs in ticks parasitizing NKGs. In total, 107 hard ticks, including *Haemaphysalis longicornis*, *Ixodes nipponensis*, and *Haemaphysalis flava*, were obtained from NKGs in South Korea between 2016 and 2019. In 40 tested tick pools, genes for four TBPs, namely *Coxiella*-like endosymbiont (CLE, 5.0%), *Candidatus* Rickettsia longicornii (45.0%), *Anaplasma bovis* (2.5%), and *Theileria luwenshuni* (5.0%) were detected. *Ehrlichia*, *Bartonella* spp., and severe fever with thrombocytopenia syndrome virus were not detected. To our knowledge, this is the first study to report CLE and *T. luwenshuni* in *H. flava* ticks in South Korea. Considering the high prevalence of *Candidatus* R. longicornii in ticks parasitizing NKGs, there is a possibility of its transmission from ticks to animals and humans. NKG ticks might be maintenance hosts for TBPs, and we recommend evaluation of the potential public health threat posed by TBP-infected ticks.

## 1. Introduction

Ticks are considered the main arthropod vectors for infectious disease agents, and they are related to important veterinary and medical health issues [1]. Numerous emergent tick-borne pathogens (TBPs) had been circulating in animals and ticks long before their detection as causes of clinical diseases [2]. Although TBPs are preserved in stable natural cycles that involve domestic and/or wild animals and ticks, humans might be considered accidental hosts [3].

The control of transmission of zoonotic pathogens with regard to vertebrate hosts and ticks, which are related in a continually changing environment, is usually difficult because of habitat distributions, zoonotic/domestic hosts, and transmission cycles in regions with coexistence of pastured domestic animals and wild animals [4]. The global hazard of TBPs is continuing to increase and is raising public health worries, with the constant identification of new pathogens over the past 20 years [5].

Understanding the ecology of local tick species and recognizing TBPs have high health significance. There is a rapidly increasing number of reservoir-adapted pathogens among various species of ticks, which are known or suspected vectors of zoonotic TBPs. Previous studies have investigated the presence of TBPs in South Korea by assessing ticks and cattle, using molecular methods [6,7,8,9,10,11,12,13]. The native Korean goat (NKG, *Capra hircus coreanae*) is an individual breed of goat primarily raised for natural health supplements and meat in South Korea, and it is known to be an essential natural reservoir host of several TBPs that influence human, wildlife, and domestic animal populations [14,15]. As NKGs are reared near domestic animals, they can transmit TBPs to these animals. However, the importance of TBPs in ticks from small ruminants, such as NKG, in South Korea is not yet known. Additionally, TBP infections in goats may be neglected because of their low economic position in the NKG production industry in South Korea. Thus, the purpose of the present study was to investigate TBPs in ticks from NKGs and assess the molecular characteristics of several TBPs.

In this study, TBP surveillance was conducted to detect tick species parasitizing goats, and to identify related tick-borne zoonotic pathogens possibly negatively influencing medical health and veterinary medicine in South Korea. The study assessed the prevalence, risk factors, and co-infectivity of TBPs, including rickettsiae (*Anaplasma*, *Ehrlichia*, and *Rickettsia*), *Bartonella* spp., *Coxiella burnetii*, piroplasms (*Babesia* and *Theileria*), and severe fever with thrombocytopenia syndrome virus (SFTSV), among ticks from NKGs in South Korea.

## 2. Results

### 2.1. Identification of Ticks

**Figure 1:**
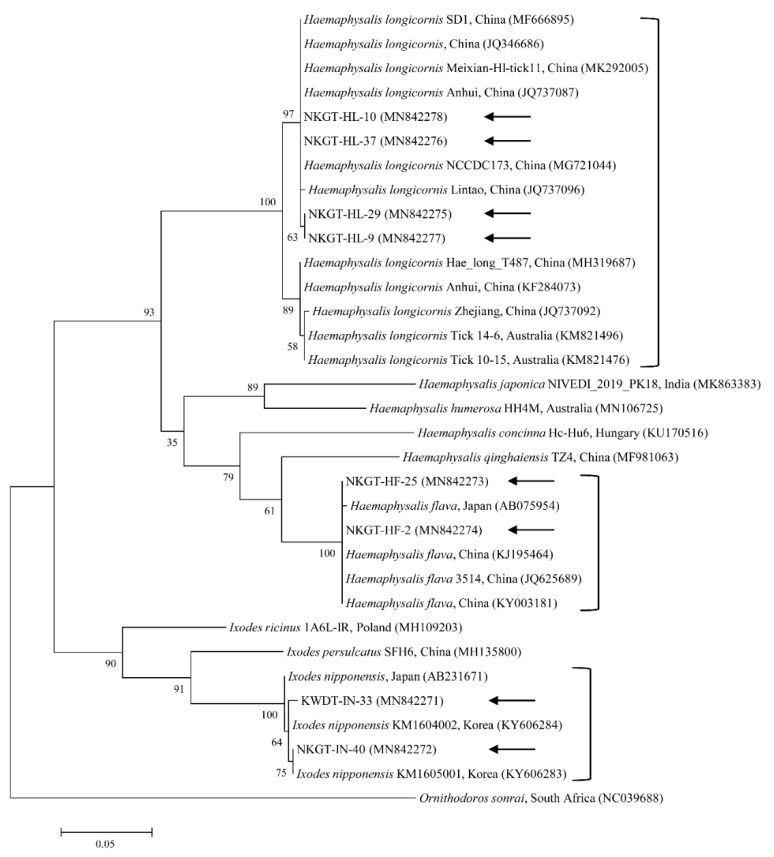
Molecular tick identification according to phylogenetic analysis using the maximum likelihood method with the mitochondrial cytochrome c oxidase subunit I gene. The analyzed sequences are indicated by arrows. The accession numbers for nucleotide sequences from GenBank are presented with species names and countries. The outgroup was *Ornithodoros sonrai*. The branch numbers mean bootstrap support (1000 replicates). The scale bar means phylogenetic distance.

We obtained 107 ticks (40 tick pools) involving two genera and three species (*Haemaphysalis longicornis*, *Ixodes nipponensis*, and *Haemaphysalis flava*) from NKGs. In the tick samples, we used universal primers for the mitochondrial cytochrome c oxidase subunit I (*cox1*) gene to amplify 710-bp fragments. The *cox1* gene was obtained and sequenced from representative ticks to eliminate potential issues in identification, especially among nymphs and larvae. In this study, the *cox1* gene sequences were divided into three groups according to nucleotide identity. The three groups shared close genetic relationships with *H. longicornis* (98.7–100% nucleotide identity), *H. flava* (98.8–100% nucleotide identity), and *I. nipponensis* (99.2–100% nucleotide identity). A phylogenetic tree was created according to the *cox1* genes of several ticks deposited in GenBank, and the collected ticks were divided into three clades related to the following three species (Figure 1): *H. longicornis* (62.5%, 25/40 pools), *H. flava* (25.0%, 10/40 pools), and *I. nipponensis* (12.5%, 5/40 pools) (Table 1).

### 2.2. Identification of TBPs

The 16S rRNA sequences of *Anaplasma bovis* (1/40 pools, 2.5%), *Coxiella*-like endosymbiont (CLE) (2/40 pools, 5.0%), and unidentified *Rickettsia* (18/40 pools, 45.0%) were detected in the ticks (Table 1). Additionally, the 18S rRNA sequence of *Theileria luwenshuni* (2/40 pools, 5.0%) was detected in the ticks (Table 1). Among the positive samples, additional genetic analysis revealed that the ticks were positive for unidentified *Rickettsia* citrate synthase (*glt*A) (18/40 pools, 45.0%). 

With regard to infection, one (2.5%) tick was co-infected with *T. luwenshuni*, CLE, and unidentified *Rickettsia*, and one (2.5%) was co-infected with *A. bovis* and *T. luwenshuni*. *Ehrlichia* spp., *Bartonella* spp., and SFTSV were not detected in this study.

With regard to each pathogen, one (6.7%) *H. longicornis* nymph was positive for *A. bovis*; one (6.7%) *H. longicornis* nymph and one (25.0%) *H. flava* adult were positive for *T. luwenshuni*; one (6.7%) *H. longicornis* nymph and one (25.0%) *H. flava* adult were positive for CLE; and eight (53.3%) *H. longicornis* nymphs and 10 (100%) *H. longicornis* adults were positive for unidentified *Rickettsia*.

### 2.3. Molecular and Phylogenetic Analyses

**Figure 2:**
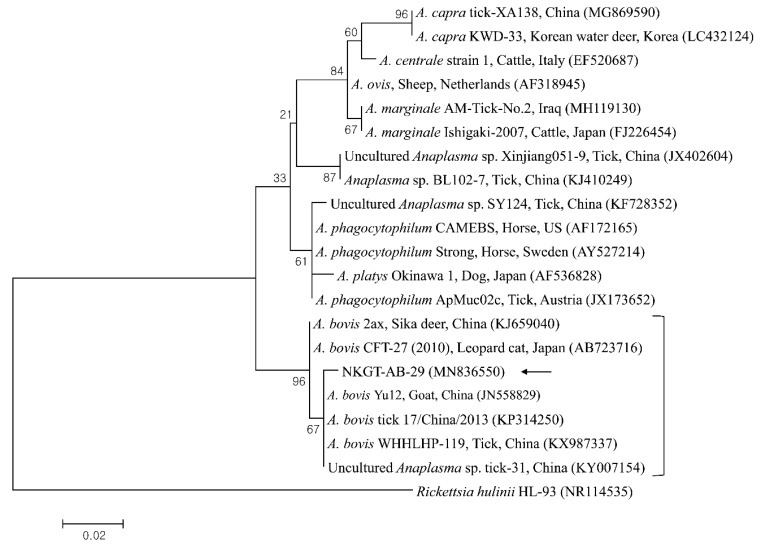
Phylogenetic tree created with the maximum likelihood method and based on *Anaplasma* spp. 16S rRNA nucleotide sequences. The analyzed sequence is indicated by an arrow. The accession numbers for nucleotide sequences from GenBank are presented with species names and countries. The outgroup was *Rickettsia hulinii*. The branch numbers mean bootstrap support (1000 replicates). The scale bar means phylogenetic distance.

**Figure 3:**
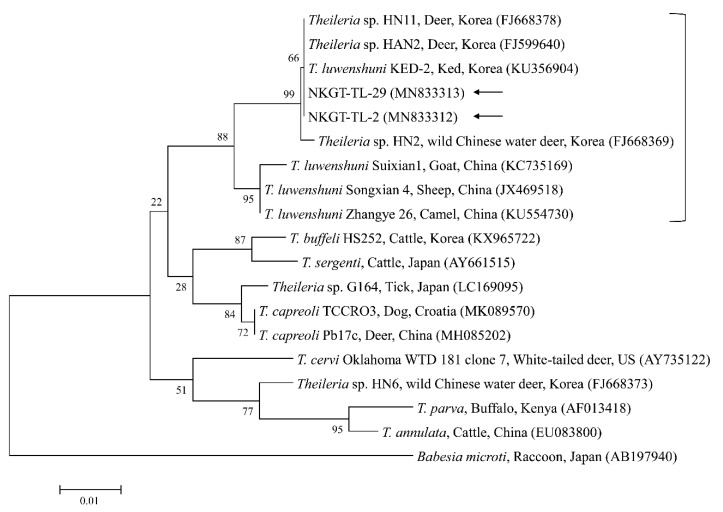
Phylogenetic tree created with the maximum likelihood method and based on *Theileria* spp. 18S rRNA nucleotide sequences. The analyzed sequences are indicated by arrows. The accession numbers for nucleotide sequences from GenBank are presented with species names and countries. The outgroup was *Babesia microti*. The branch numbers mean bootstrap support (1000 replicates). The scale bar means phylogenetic distance.

**Figure 4:**
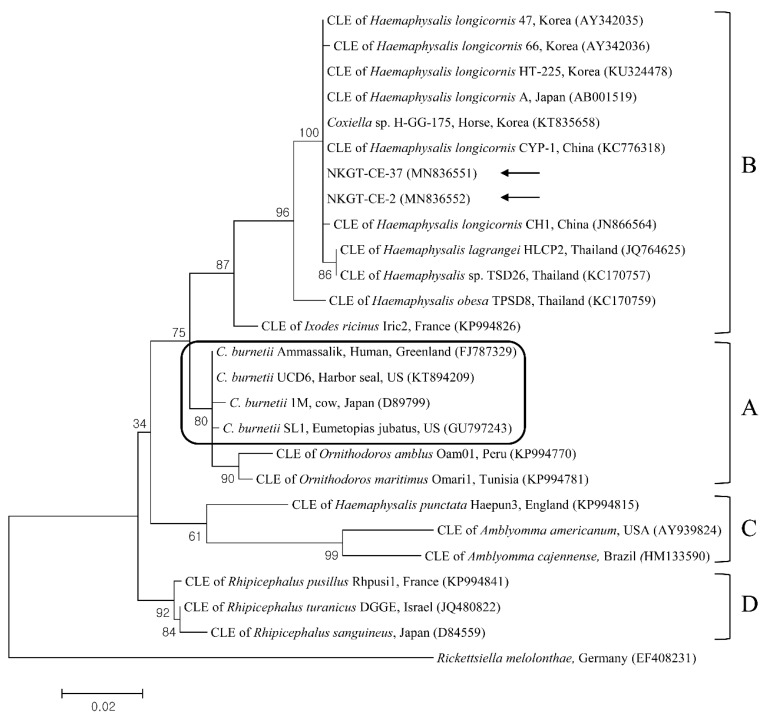
Phylogenetic tree created with the maximum likelihood method and based on *Coxiella* spp. 16S rRNA nucleotide sequences. The outgroup was *Rickettsiella melolonthae*. The analyzed sequences are indicated by arrows. Four clades of *Coxiella* are indicated as A to D. The *Coxiella burnetii* group is outlined within clade A. The accession numbers from GenBank for other sequences are presented with sequence names. The branch numbers mean bootstrap support (1000 replicates). The scale bar means phylogenetic distance. CLE = *Coxiella*-like endosymbionts.

**Figure 5:**
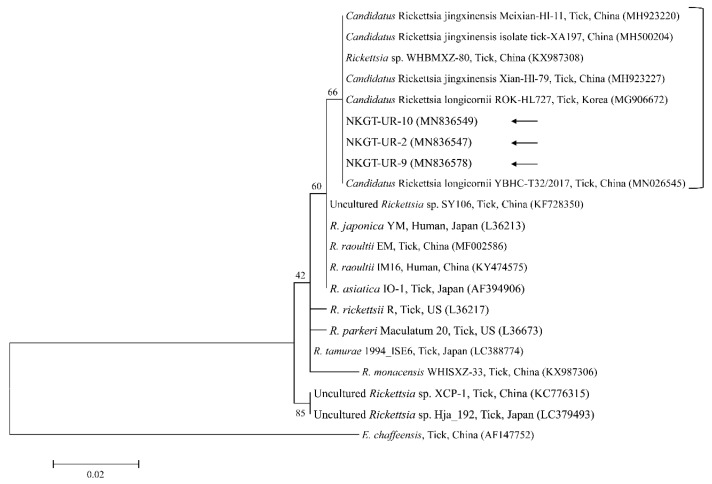
Phylogenetic tree created with the maximum likelihood method and based on *Rickettsia* spp. 16S rRNA nucleotide sequences. The analyzed sequences are indicated by arrows. The accession numbers for nucleotide sequences from GenBank are presented with species names and countries. The outgroup was *Ehrlichia chaffeensis*. The branch numbers mean bootstrap support (1000 replicates). The scale bar means phylogenetic distance.

**Figure 6:**
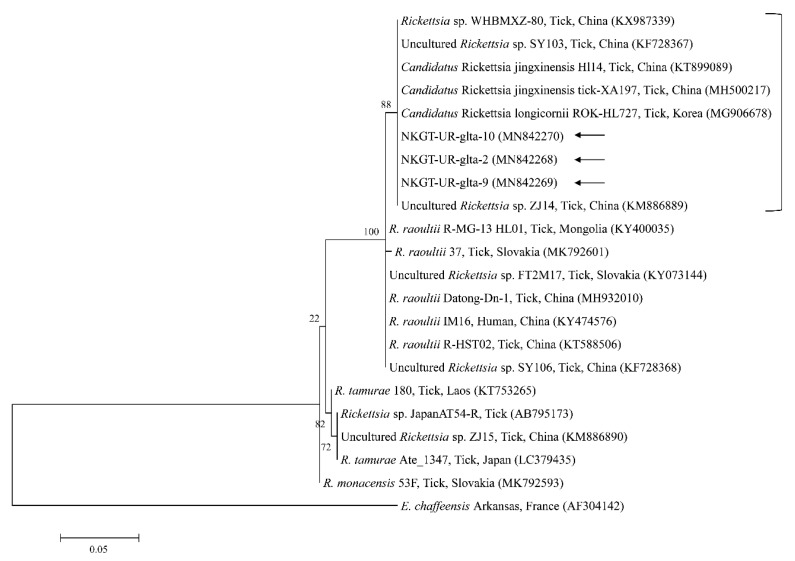
Phylogenetic tree created with the maximum likelihood method and based on *Rickettsia* spp. *glt*A nucleotide sequences. The analyzed sequences are indicated by arrows. The accession numbers for nucleotide sequences from GenBank are presented with species names and countries. The outgroup was *Ehrlichia chaffeensis*. The branch numbers mean bootstrap support (1000 replicates). The scale bar means phylogenetic distance.

In phylogenetic analyses, the 16S rRNA sequences of *A. bovis* (Figure 2), 18S rRNA sequences of *T. luwenshuni* (Figure 3), 16S rRNA sequences of *Coxiella* (Figure 4), and 16S rRNA (Figure 5) and *glt*A (Figure 6) nucleotide sequences of *Rickettsia* were clustered with previously documented sequences.

One sequence of *A. bovis* showed 98.8–99.5% identity with 16S rRNA sequences in previously reported *A. bovis* isolates. Two sequences of *T. luwenshuni* shared 100% identity with 18S rRNA sequences. They also shared 97–100% identity with 18S rRNA sequences in previously reported *T. luwenshuni* isolates. Two sequences of CLE shared 100% identity with 16S rRNA sequences. They also shared 98.4–100% identity with 16S rRNA sequences in previously reported CLE clade B isolates.

Three representative sequences of unidentified *Rickettsia* genes shared 100% identity with 16S rRNA and *glt*A sequences. They also shared 99.8–100% identity with 16S rRNA sequences and 97.6–99.2% identity with *glt*A sequences in previously reported unidentified *Rickettsia* isolates.

The identified representative sequences were submitted to GenBank. The accession numbers are as follows: MN842271–MN842272 (*I. nipponensis*), MN842273–MN842274 (*H. flava*), MN842275–MN842278 (*H. longicornis*), MN836550 (*A. bovis*), MN833312–MN833313 (*T. luwenshuni*), MN836551–MN836552 (CLE), MN836547–MN836549 (unidentified *Rickettsia* 16S rRNA), and MN842268–MN842270 (unidentified *Rickettsia glt*A).

## 3. Discussion

The present study found *H. longicornis* (62.5%), *H. flava* (25.0%), and *I. nipponensis* (12.5%) in NKGs, and the most common species was *H. longicornis*. These findings are similar to the results of a previous South Korean study, which found 569 *H. longicornis* organisms (38 nymphs, 369 male adults, and 162 female adults) from 27 goat farms [16].

The *cox1* sequences from collected *H. longicornis* showed 97.1–99.6% nucleotide identity with known *cox1* sequences of *H. longicornis* (Figure 1). The *cox1* sequences from collected *H. flava* and *I. nipponensis* showed 98.3–98.7% and 98.2–99.8% nucleotide identity with known *cox1* sequences, respectively (Figure 1). *H. longicornis* is mainly obtained from grasses and herbaceous vegetation, *H. flava* is frequently obtained from forests, and *I. nipponensis* is obtained from both habitats across South Korea [17]. All three ticks identified in this study are vectors and hosts for many TBPs and can transmit them to animals and humans in South Korea [6,7,8,9,10,11,12,13].

Several TBPs, including *A. bovis*, *T. luwenshuni*, CLE, and unidentified *Rickettsia*, were detected in NKG ticks via molecular analysis. The influences of diseases associated with *Anaplasma* regarding the health and productivity of animals have been known for over a century, and there is a major threat to industries related to livestock [18]. In nature, *Anaplasma* movement involves tick vectors, and there are many vertebrate hosts and infection sources for ticks, animals, and humans [18]. *A. bovis*, which is a monocytotropic species, has been identified among ruminants in numerous countries [19]. *A. bovis* is not considered a zoonotic pathogen, and it could be identified in cattle having subclinical signs, including lymphadenopathy, depression, fever, and abnormal conditions [20]. *A. bovis* was previously detected in South Korea in ticks (7.5%, 20/266 pools) from Korean water deer [7], in cattle (1.0%, 12/1219) [10], and in *H. longicornis* ticks (1.0%, 5/506 pools) [6]. Additionally, it has been detected in *H. longicornis* ticks (26.4%, 77/292) from goats in North Korea [8]. Moreover, *Anaplasma* spp. have been detected in NKGs (29.2%, 7/39) [15]. In the present study, one *H. longicornis* nymph from an NKG was positive for *A. bovis*.

Theileriosis, which is a tick-borne hemoprotozoan disease, can affect domestic animals, most frequently sheep and cattle in tropical and sub-tropical areas, and it causes high economic loss [21]. *T. luwenshuni* mainly infects small ruminants and is transmitted by *H. longicornis* [22]. In South Korea, *T. luwenshuni* has been previously identified in roe deer (100%, 23/23), and *H. longicornis* (34.8%, 8/23 pools) [11], and in deer keds from Korean water deer (75%, 6/8) [23]. In this study, two ticks (5.0%) from NKGs tested positive for *T. luwenshuni* (one *H. longicornis* nymph and one *H. flava* adult). To our knowledge, the present study is the first to report the presence of *T. luwenshuni* in *H. flava* ticks in Korea.

Q fever, which is caused by *C. burnetii* and is a zoonosis, has a global distribution, and it can result in a severe condition in animals and humans [24]. The biological features of CLE differ from those of *C. burnetii*, and some of the organisms might behave like symbionts that are engaged in complex interactions with ticks. It is considered that CLE are closely related to, but genetically distinct from, *C. burnetii*, suggesting extensive diversity within *Coxiella*. Through multilocus sequencing, *Coxiella* has been divided into at least four extremely divergent genetic clades (A to D), and *C. burnetii* has been categorized within clade A [25]. In South Korea, *C. burnetii* was previously identified in NKGs (9.5%, 57/597) [14], and CLE clade B was identified in *H. longicornis* ticks (52.4%, 121/213) from horses [9]. In this study, two ticks (5.0%) from NKGs tested positive for CLE clade B (one *H. longicornis* nymph and one *H. flava* adult). To our knowledge, the present study is the first to report the presence of CLE in *H. flava* ticks in Korea. However, in this study, *C. burnetii* was not detected. Additional investigations are needed to evaluate the prevalence of infection involving *C. burnetii* or CLE among ticks and NKGs.

Organisms from the genus *Rickettsia* have been divided into various groups, including the typhus group, spotted fever group (SFG), *Rickettsia canadensis* group, and *Rickettsia bellii* group [26]. Obligate intracellular bacteria belonging to the SFG cause tick-borne rickettsioses [27]. The significance and scope of recognized tick-borne rickettsial pathogens have greatly increased in the last 25 years, and thus, this disease complex is considered an ideal model for recognizing emerging and re-emerging infections [5]. In this study, one SFG rickettsia with *Candidatus* status was detected. A possibly novel SFG rickettsia classified into the putative new subgroup “*Candidatus* Rickettsia longicornii” was identified in the species *H. longicornis* (100%, 11/11 pools; 43.2%, 79/183) in South Korea [13]. These strains were clustered in a subcategory that represented a sister taxon separate from the known subcategories of SFG rickettsiae. Sequences of gene fragments in GenBank for rickettsial isolates obtained from *H. longicornis* in Japan, China, and South Korea have unknown pathogenicity and uncertain taxonomic statuses that are likely associated with *Candidatus* R. longicornii or a closely connected species [13]. The others were given provisory species names, including *Candidatus* Rickettsia jingxinensis from *H. longicornis* (1.0%, 2/204 pools) in China [28]. The two uncultured *Rickettsia* species were found to be the same species by phylogenetic analysis involving several genes [29]. In a previous study, unidentified new *Rickettsia* spp. in *H. longicornis* were *Candidatus* R. longicornii (16.7%, 52/311 pools) in South Korea [12] and *Candidatus* R. jingxinensis (32.0%, 303/947) in China [29]. Moreover, the XY118 (KU853023) isolate was obtained from a human, indicating its possible pathogenicity in humans. Additional studies are required to determine its pathogenicity. In the present study, unidentified *Rickettsia* spp. (45.0%) were detected in eight *H. longicornis* nymphs and 10 *H. longicornis* adults, and the pathogen was identified as *Candidatus* R. longicornii by phylogenetic analysis. Further surveys are needed to identify other novel *Rickettsia* spp. in ticks and their host animals.

The bootstrap support values need to be analyzed carefully. As a limitation in this study, some bootstrap values were low. This could be due to using small amplification fragments for phylogeny. Thus, an additional study is needed to analyze longer fragments of genes in phylogeny for better presentation of data.

In conclusion, four TBPs were identified in ticks obtained from NKGs in South Korea, including zoonotic pathogens of CLE and *Candidatus* R. longicornii. To the best of our knowledge, this is the first report of CLE and *T. luwenshuni* in *H. flava* ticks from South Korea. Additionally, there is a high prevalence of *Candidatus* R. longicornii in ticks parasitizing NKGs. Both local inhabitants of NKG farms and animals are at high risk of exposure to coxiellosis, anaplasmosis, theileriosis, and rickettsiosis. The present findings extend our knowledge of the distribution and probable vector spectrum of TBPs and suggest that ticks are a potential reservoir for TBP transmission to other animals and humans via bites. Investigation of the tick infestation risk is important, especially from the zoonotic and public health perspectives. These data associated with a high TBP prevalence in ticks indicate the requirement for further studies on the impacts of tick-borne diseases in animals and humans. Better understanding of local major tick species and thorough TBP characterization are essential to public health. Limited information is available about the TBPs of ticks from small ruminants, such as NKG, in South Korea. Although limited numbers of ticks were collected from goats in this study, tick abundance and distribution patterns were similar to those in previous studies [7,17], showing predominance of *H. longicornis*, followed by *H. flava* and *I. nipponensis*. Thus, we believe that the present findings extend our knowledge of the distribution and probable vector spectrum of TBPs.

## 4. Materials and Methods

### 4.1. Tick Collection and Species Identification

In 2018, the total number of NKGs reared in South Korea was reported to be 542,744 from 14,644 farms [30]. NKGs are mostly reared on small-scale farms and are allowed to graze freely in the pasture and mountain areas. These farms are in distant places. These factors were considered to hinder tick sampling. A total of 107 ticks were collected from NKGs in South Korea using a simple random sampling method by veterinarians at local veterinary institutions during treatment, surveillance, and monitoring or during regular check-up after receiving oral approval from NKG farm owners between 2016 and 2019. Two to ten ticks per NKG were collected from 19 NKGs, and they were preserved in 70% ethanol. The collected ticks were initially identified according to their morphological characteristics [31], with further classification according to the molecular methods described below. Subsequently, the ticks were pooled by species and developmental stage (nymph/adult) into 40 tick pools, with one to three ticks per pool.

### 4.2. Molecular Detection of Ticks and TBPs

A commercial DNeasy Blood & Tissue Kit (Qiagen, Melbourne, Australia) was used according to the provided instructions for genomic DNA extraction from the ticks. The extracted DNA was kept at −20 °C. Polymerase chain reaction (PCR) amplification was conducted using the AccuPower^®^ ProFi Taq PCR Premix Kit (Bioneer, Daejeon, South Korea). Molecular identification of tick species was conducted by amplifying the sequence of the *cox1* gene using specific primers [32].

The ticks were then screened for several TBPs with primers specific to each pathogen. The presence of rickettsiae was initially assessed by PCR with a commercial AccuPower^®^ Rickettsiales 3-Plex PCR Kit (Bioneer) for the detection of rickettsiae 16S rRNA. For species identification, positive samples were then amplified further. For *Rickettsia* spp., positive samples were confirmed by PCR that targeted *glt*A [33]. Multiple primers were used to amplify the 16S rRNA fragment for the genus *Coxiella* (including *C. burnetii* and CLE) [9]. Additionally, nested PCR (nPCR) was used to amplify the internal transcribed spacer region sequence of *Bartonella* spp. [34]. The S segment of SFTSV was amplified by nPCR [35]. Piroplasm infection was initially screened using a commercial AccuPower^®^ *Babesia* & *Theileria* PCR Kit (Bioneer) to detect piroplasm 18S rRNA. Positive samples were then re-amplified by PCR using primers designed from the common sequence of the 18S rRNA gene of some piroplasm species [36].

All primers and amplification conditions used for the detection of TBPs in ticks from NKGs in the present study are described in Supplementary Table S1.

### 4.3. DNA Cloning

Agarose gel extraction kits (Qiagen) were used to purify positive PCR products. The purified products were introduced into pGEM-T Easy vectors (Promega, Madison, WI, USA), according to the provided instructions. The ligated products were then transformed into *Escherichia coli* DH5α-competent cells (Thermo Fisher Scientific, Wilmington, DE, USA), and the cells were incubated at 37 °C overnight. Plasmid DNA was extracted using the Plasmid Miniprep Kit (Qiagen), according to the provided instructions.

### 4.4. Nucleotide Sequencing and Phylogeny

Sequencing was performed using recombinant plasmid clones. The sequences were analyzed and aligned with the multiple sequence alignment CLUSTAL Omega (v. 1.2.2) program, and the alignments were corrected with BioEdit (v. 7.0.4). Phylogeny was assessed with the maximum likelihood method and the Kimura 2-parameter distance model in MEGA (v. 5.0). Sequence analysis involved a similarity matrix. For tree stability, bootstrap analysis with 1,000 replicates was performed.

## Figures and Tables

**Table 1:** Identified tick-borne pathogens in ticks from native Korean goats in South Korea, 2016–2019.

Species	Stage	Tick Pool (n)	*A. bovis* (16S)		*T. luwenshuni* (18S)		*Coxiella*-like Endosymbiont (16S)		*Candidatus* R. Longicornii (16S)	
			Positive (%)	95% CI	Positive (%)	95% CI	Positive (%)	95% CI	Positive (%)	95% CI
*Haemaphysalis longicornis*	Nymph	15	1 (6.7)	0–19.3	1 (6.7)	0–19.3	1 (6.7)	0–19.3	8 (53.3)	28.1–78.6
	Adult	10	0	0	0	0	0	0	10 (100)	100
*Haemaphysalis flava*	Nymph	6	0	0	0	0	0	0	0	0
	Adult	4	0	0	1 (25.0)	0–67.4	1 (25.0)	0–67.4	0	0
*Ixodes nipponensis*	Nymph	3	0	0	0	0	0	0	0	0
	Adult	2	0	0	0	0	0	0	0	0
Total		40	1 (2.5)	0–7.3	2 (5.0)	0–11.8	2 (5.0)	0–11.8	18 (45.0)	29.6–60.4

16S, 16S rRNA; 18S, 18S rRNA; 95% CI, 95% confidence interval; *A*., *Anaplasma*; *T*., *Theileria*; *R*., *Rickettsia.*

## Supplementary Material

The following are available online at www.mdpi.com/xxx/s1, Table S1: Primers used for the detection of tick-borne pathogens in ticks from native Korean goats (*Capra hircus coreanae*) in the present study.
