# Supplementary material for: Molecular and Phylogenetic Analysis of Tick-Borne Pathogens in Ticks Parasitizing Native Korean Goats (Capra hircus coreanae) in South Korea
Source: Pathogens. 2020 Jan 21;9(2):71. doi: 10.3390/pathogens9020071 (PMC7168648; doi:10.3390/pathogens9020071)
Supplement: Supplementary file 1 [file pathogens-09-00071-s001.pdf]

Supplementary Table S1: Primers used for the detection of tick-borne pathogens in ticks from native Korean goats (*Capra hircus coreanae*) in the present study.

Organism	Gene	Primer	Sequence 5′ to 3′	Size (bp)	Amplification condition	Reference
Invertebrate	Mitochondrial cytochrome c oxidase subunit I	LCO1490	GGTCAACAAATCATAAAGATATTGG	710	95°C/5 min; 35 cycles: 95°C/60 s, 40°C/60 s, 72°C/30 s; 72°C/10 min	Folmer et al., 1994
		HC02198	TAAACTTCAGGGTGACCAAAAAATCA			
Anaplasma spp.	16S rRNA	*		429	95°C/5 min; 45 cycles: 95°C/30 s, 59°C/30 s, 72°C/30 s; 72°C/10 min	Commercial AccuPower® Rickettsiales 3-Plex PCR Kit (Bioneer)
Ehrlichia spp.	16S rRNA	*		340	95°C/5 min; 45 cycles: 95°C/30 s, 59°C/30 s, 72°C/30 s; 72°C/10 min	Commercial AccuPower® Rickettsiales 3-Plex PCR Kit (Bioneer)
Rickettsia spp.	16S rRNA	*		252	95°C/5 min; 45 cycles: 95°C/30 s, 59°C/30 s, 72°C/30 s; 72°C/10 min	Commercial AccuPower® Rickettsiales 3-Plex PCR Kit (Bioneer)
Rickettsia spp.	*glt*A	Rsfg877	GGGGGCCTGCTCACGGCGG	380	95°C/10 min; 35 cycles: 95°C/60 s, 51°C/60 s, 72°C/60 s; 72°C/10 min	Reis et al., 2011
		Rsfg1258	ATTGCAAAAAGTACAGTGAACA			
Coxiella spp.	16S rRNA	Cox16SF1	CGTAGGAATCTACCTTRTAGWGG	1321–1429	93°C/3 min; 30 cycles: 93°C/30 s, 56°C/30 s, 72°C/60 s; 72°C/5 min	Seo et al., 2016
		Cox16SR2	GCCTACCCGCTTCTGGTACAATT			
		Cox16SF2	TGAGAACTAGCTGTTGGRRAGT	624–627	93°C/3 min; 30 cycles: 93°C/30 s, 56°C/30 s,	

		Cox16SR2	GCCTACCCGCTTCTGGTACAATT		72°C/60 s; 72°C/5 min	
Bartonella spp.	ITS-1	QHVE-OF	TTCAGATGATGATCCCAAGC	736	94°C/10 min; 35 cycles: 94°C/60 s, 55°C/60 s, 72°C/120 s; 72°C/5 min	Ko et al., 2013
		QHVE-OR	AACATGTCTGAATATATCTTC			
		QHVE-IF	CCGGAGGGCTTGTAGCTCAG	484		
		QHVE-IR	CACAATTTCAATAGAAC			
Severe fever with thrombocytopenia syndrome virus	S segment	NP-2F	CATCATTGTCTTTGCCCTGA	461	50°C/30 min; 95°C/15 min; 40 cycles: 95°C/20 s, 52°C/40 s, 72°C/30 s; 72°C/5 min	Yoshikawa et al., 2014
		NP-2R	AGAAGACAGAGTTCACAGCA			
		N2F	AAYAAGATCGTCAAGGCATCA	346	25 cycles: 94°C/20 s, 55°C/40 s, 72°C/30 s	
		N2R	TAGTCTTGGTGAAGGCATCTT			
Babesia spp. and *Theileria* spp.	18S rRNA	*		676	95°C/5 min; 35 cycles: 95°C/30 s, 59°C/30 s, 72°C/30 s; 72°C/5 min	Commercial AccuPower® Babesia & Theileria PCR Kit (Bioneer)
Babesia spp. and *Theileria* spp.	18S rRNA	BJ1	GTCTTGTAATTGGAATGATGG	452	95°C/5 min; 40 cycles: 94°C/30 s, 54°C/30 s, 72°C/40 s; 72°C/5 min	Casati et al., 2006
		BN2	TAGTTTATGGTTAGGACTACG			

* Commercial PCR kits were used for the detection of these pathogens.
